# Afternoon kick-off, evening kick-off, or night kick-off in the first German Bundesliga – A possible Injury risk factor?

**DOI:** 10.1051/sicotj/2024049

**Published:** 2024-11-26

**Authors:** Erik Schiffner, Dominique Schoeps, Christos Koukos, Felix Lakomek, Joachim Windolf, David Latz

**Affiliations:** 1 Department of Orthopedics and Trauma Surgery, Heinrich Heine University Hospital Duesseldorf Moorenstr. 5 40225 Duesseldorf Germany; 2 Sports Trauma and Pain Institute 196 Vasilissis Olgas Avenue, 27 Ploutonos Street 54655 Thessaloniki Greece

**Keywords:** Soccer, Kick-off time, Sports medicine

## Abstract

*Introduction*: This retrospective cohort study aimed to evaluate the impact of kick-off time on the risk of injury for professional soccer players in the first German Bundesliga. It was hypothesized that late kick-off times would have a negative effect on muscle and ligament injuries to the ankle and knee. *Methods*: Kick-off times and injury data were collected over 5 consecutive seasons (1530 matches; 2014–2019) from two media-based registries (transfermarkt.de^®^ und kicker.de^®^). The kick-off times were assorted into three groups: Afternoon kick-off between prior to 3:30 pm (988 matches), evening kick-off between 5:30 to 6:30 pm (303 matches), and night kick-off after 8 pm (239 matches). *Results*: A total of 1327 match injuries were recorded over 5 seasons in 510 different male elite soccer players. The injuries affected muscles in 32.1%, ankle ligaments in 7.8%, and knee ligaments in 5.6%. There was no significant difference in injury rates when comparing different kick-off time groups (*p* > 0.05), however, the mean of time attributed to muscle and ankle ligament injuries suffered in games with a late kick-off time was significantly longer (*p* < 0.05). *Conclusion*: This study shows that there is no significant (*p* > 0.05) association between three different kick-off time groups and injury risk in the first German Bundesliga. However, significant (*p* < 0.05) differences in the lay-off times attributed to muscle and ankle ligament injuries differed with different kick-off times assorted into the three groups. Reasons for this observation could be found in the circadian muscle rhythms and muscle fatigue.

## Introduction

The physical load on professional soccer players is high [1]. In soccer strength, speed, agility, and endurance are considered important components of a player’s performance [2]. The mean total distance covered during a soccer match is reported to be between 10 and 11 km, with some players covering up to 14 km in a 90-minute match [3, 4]. This includes 150–250 high-intensity sprints for each player during a professional soccer game [5]. It has been shown that it takes several days to fully recover from this effort [6].

In recent years, several studies evaluated incidences and patterns of injuries in professional soccer players [7, 8]. It has been shown, that injury rates are higher in matches than in practice [9]. Also, Pfirrmann et al. have reported, that most lower extremity injuries in soccer affect the knee and ankle joint, as well as the hamstring muscles [9]. Ekstrand et al. [10] reported a higher incidence of knee joint and muscle injuries suffered in the final stages of a soccer game and attributed this to increased muscle fatigue. It is reasonable to suppose that physiological fatigue accumulates during a season of intense practice and multiple matches in elite soccer.

Various studies have focused on physiology, biomechanics, performance, fatigue, and injury risk factors in elite soccer players [5, 11–14]. However, there is no study that has investigated the impact of kick-off time as a risk factor for injuries. In the first German Bundesliga, there are various kick-off times ranging from 1:30 pm to 8:30 pm.

The purpose of the present study was to determine the effect of different kick-off time groups on the risk of injury in the first German Bundesliga. We hypothesized that due to circadian rhythm, a late kick-off time would have a negative effect on muscle and ligament injuries in professional soccer players.

## Material and methods

### Study design

The study was designed as a retrospective cohort study. Included were all male soccer players assigned to one of the 18 teams of the first German Bundesliga from July 2014 until June 2019. Non-professional players without a contract were not included. The average age was 23.9 years.

### Data collection

All data used in this study was retrieved from kicker.de^®^ and transfermarkt.de^®^. These are two open-source sports databases containing information on injuries of players in a longitudinal manner. Data from all teams of the 1st Bundesliga from the season of 2014/2015 to the season of 2018/19 was analyzed. The kick-off times and the history of injuries are available in a charted form as part of each player’s technical details. This includes reports of kick-off times, the start and end date of injuries, and the type and localization of the injury. The study was approved by the local ethics review board. Written informed consent was collected from kicker.de and transfermarkt.de.

### Injury definition

All injuries that occurred during a competition in the First German Bundesliga were reported and collected. A match injury was defined if the player was unable to participate in training or competition for at least one day beyond the day of injury. Injuries were considered as a “match injury” if reported on the same day or the following day of a match in that the player was actively participating [8]. Return to soccer was defined as the number of days from injury to unlimited soccer training with the team. A muscle injury was defined as a traumatic distraction or overuse injury to a muscle [15] and a ligament injury (ankle, knee) was defined as an acute distraction injury of ligaments or joint capsules or ligament ruptures [16].

### Statistical analysis

Statistical analysis in this study was conducted with the support of a biostatistician who used the SPSS software pack (version 23, IBM, New York, USA). The player’s exposure was calculated based on the actual time participating in official matches during the season. Chi-squared tests were used to compare the incidences between the three kick-off time groups. One-way ANOVA (analysis of variance) was performed to compare mean injury-related lay-off times between the three kick-off time groups. A *p*-value < 0.05 was regarded as a statistically significant difference.

## Results

### General

A total of 1327 match injuries in 510 different male elite soccer players were recorded in the 5 seasons observed. The demographic data of these players is shown in Table 1.

**Table 1:** Match injuries demographics, *n* = match injuries.

Season	All players	Mean age	*n*	*n* per 100 players 95% CI
2014/15	574	23.7	155	27.0 (22.3–31.2)
2015/16	602	23.7	231	38.4 (31.4–42.4)
2016/17	644	23.9	290	45.0 (39.5–48.3)
2017/18	600	24.0	316	52.7 (48.3–56.1)
2018/19	633	24.0	335	52.9 (48.8–56.5)

During the period observed the First German Bundesliga had eight different kick-off times. For a clear arrangement, these kick-off times were assorted into three groups: Afternoon kick-off between prior to 3:30 pm (988 matches), evening kick-off between 5:30 to 6:30 pm (303 matches), and night kick-off after 8 pm (239 matches).

Table 2 demonstrates the number of match injuries and the mean of time according to the kick-off times.

**Table 2:** Kick-off times, matches, injuries, mean off time (days).

Kick-off time	Matches	Injuries	Injury/Match	Mean off days
1:30 pm	10	10	1	15
3:30 pm	978	822	0.84	26.2
	988	832	0.84	20.6
5:30 pm	92	67	0.72	23.7
6:00 pm	61	68	1.11	18.6
6:30 pm	150	126	0.84	20.7
	303	261	0.86	21.0
8:00 pm	72	42	0.59	26.4
8:15 pm	1	3	3	40.7
8:30 pm	166	189	1.14	30.1
	239	234	0.97	32.4

There was no significant difference in injury rates when comparing the three different kick-off time groups (*p* > 0.05). The overall mean off time after a match injury was moderate with 23.2 days (range 1–377 days). There was a significant difference in the overall off times between the afternoon and evening groups compared to the night group (*p* < 0.05). The 1327 injuries involved the muscle in 32.1%, the ankle ligaments in 7.8% and the knee ligaments in 5.6%.

#### Muscle injuries

There were 427 muscle injuries recorded. Overall there was no significant difference in the incidence of muscle injuries for the three different kick-off time groups. The mean off time after these injuries was 24.1 days (range 1–294 days). When comparing the three groups (Table 3) the mean of time was significantly longer in the night group (*p* < 0.05).

**Table 3:** Kick-off times, matches, muscle injuries, mean off time (days).

Kick-off time	Matches	Injuries	Injury/Match	Mean off time
1:30 pm	10	3	0.3	9
3:30 pm	978	255	0.26	18.9
	988	258	0.26	13.9
5:30 pm	92	18	0.19	14.7
6:00 pm	61	22	0.36	23.2
6:30 pm	150	41	0.27	14.7
	303	81	0.27	17.5
8:00 pm	72	18	0.25	30.7
8:15 pm	1	2	2	59
8:30 pm	166	68	0.41	22.5
	239	88	0.36	37.4

#### Ankle ligament

There was no significant difference between kick-off time groups and the rate of ankle ligament injuries (*p* > 0.05). However, a significant correlation was seen in the duration of the injury-related lay-off between the afternoon and evening group compared to the night group (*p* < 0.05) (Table 4).

**Table 4:** Kick-off times, matches, ankle ligament injuries, mean off time (days).

Kick-off time	Matches	Injuries	Injury/Match	Mean off time
1:30 pm	10	0	0	0
3:30 pm	978	60	0.06	42.6
	988	60	0.06	21.3
5:30 pm	92	8	0.09	31
6:00 pm	61	5	0.08	27.8
6:30 pm	150	6	0.04	15.3
	303	19	0.06	24.7
8:00 pm	72	3	0.04	50.7
8:15 pm	1	0	0	0
8:30 pm	166	15	0.09	55.7
	239	18	0.08	35.5

#### Knee ligaments

There was no significant association between the three kick-off time groups and the incidence of ligamentous injuries of the knee (*p* > 0.05). However, the mean layoff time after knee ligament injuries was significantly shorter in the night group compared to the afternoon and evening groups (*p* < 0.05) (Table 5).

**Table 5:** Kick-off times, matches, knee ligament injuries, mean off time (days).

Kick-off time	Matches	Injuries	Injury/Match	Mean off time
1:30 pm	10	1	0.1	38
3:30 pm	978	52	0.05	89.5
	988	53	0.05	63.7
5:30 pm	92	3	0.03	65
6:00 pm	61	2	0.03	32
6:30 pm	150	10	0.07	60.3
	303	15	0.05	52.4
8:00 pm	72	0	0	0
8:15 pm	1	0	0	0
8:30 pm	166	6	0.04	86.8
	239	6	0.02	28.9

## Discussion

Overall, no significant associations were found between afternoon, evening, or night kick-off and the incidence of an injury in matches of the first German Bundesliga. However, this study shows a significant difference in the suspected injury severity for different kick-off time groups.

Reasons why no associations in the incidence of injuries were observed could be the rotation of players in the roster of a team and other effective prevention strategies for fatigue of individual players [17, 18]. Dupont et al. [17] observed a significantly higher injury rate when players attended two matches per week compared to one match a week. The authors suggested a time interval for recovery of 72–96 h between two matches or the rotation of players from the roster to reduce the injury rate. In the first German Bundesliga, an average of 22 players are required on the roster of a team (transfermarkt.de^®^). This way for all positions a duplicate player is available and the teams will be able to rotate individual players in their starting line-up [1].

Interestingly a significant association of the lay-off times following muscle injuries to the three kick-off time groups was seen. The lay-off time serves as an indirect measure of the injury severity. The mean lay-off time for muscle injuries was significantly longer when suffered in night matches compared to matches that started in the afternoon or evening. One reason for this observation could be the circadian rhythm and its impact on muscle fatigue [19–22]. The relationship between circadian rhythm and skeletal muscle function and fatigue was the subject of previous studies [21, 22]. More than 2300 genes in skeletal muscle are expressed in a circadian pattern, and these genes participate in a wide range of functions, including myogenesis, transcription, and metabolism [22]. This highlights the role of the circadian rhythms in muscle structure, function, and metabolism [22]. A lot of studies have shown that skeletal muscle torque, strength, and power are higher in the late afternoon [23]. Some authors divide individuals into three groups, early circadian phenotype (ECT), intermediate circadian phenotype (ICT), and late circadian phenotype (LCT) [24]. Hence the highest performances are obtained at different times of the day, i.e. 12:00 for ECT, 16:00 for ICT, and 19:00 for LCT [24]. This given factor could be accountable for differences observed in the incidence and severity of injuries observed. Even though no differences in the incidence of injuries were seen, the mean absence from soccer following a muscle injury suffered in night matches was significantly higher compared to the afternoon- or evening matches. Also, previous studies have shown that fatigue during a football match is a potential cause of muscle injuries in professional soccer. These injuries are more common approaching the end of halves [20]. Also, another type of fatigue has been suggested: Two studies show a higher incidence of injuries when players attend two matches compared to one match per week [1, 17].

The mean lay-off time following ankle injuries suffered in night matches was significant longer compared to early kick-off time groups. Some of the main stabilizers of the ankle joint are the peroneus and the tibialis posterior muscle [25]. As mentioned above, the circadian rhythm could induce a lack of muscle stabilization of the ankle joint which may lead to ligamentous injuries.

**Figure 1:**
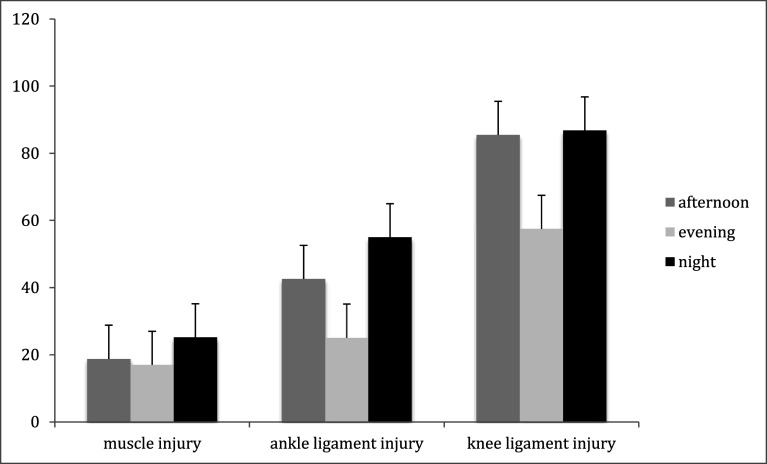
Mean off times (days).

An association between the three kick-off time groups and mean lay-off times following ligamentous injuries of the knee has been observed, as well (Figure 1). Injuries suffered in afternoon- and evening matches led to longer lay-off times compared to night matches. This is in contrast to the findings for other injuries. However, the correlation between injury severity and lay-off time is somewhat diminished in these injuries. Whereas the vast majority of ligamentous injuries to the ankle and muscle injuries are subjected to non-operative treatment [26], in cases of severe ligamentous injuries to the knee surgical reconstruction is now considered the gold standard [27]. Hence there is a multitude of influencing factors on the lay-off time, such as the method of surgical treatment, complications, and the rehabilitation protocol. However, the contradictory association with the time of the day and injury lay-off time following ankle and knee injuries remains nebulous, and further studies are needed to investigate these correlations.

The main limitation of this study is that it is based on media information. This data is not validated with medical records by each team’s medical departments, but it is published by non-medical professionals and club staff. Thus, this data should be interpreted with caution [28]. However, media databases have been used previously to analyze correlations to injuries in professional soccer [28–30]. Also, information obtained from medical records, the considered gold standard for research purposes, is not guaranteed to be flawless. Bjorneboe et al. [31] showed that medical staff reporting failed to capture about 20% of all injuries leading to layoff times. However, by the extensive media coverage of the first German Bundesliga, a comprehensive reporting system of all relevant information has been established. This study has other limitations, such as its retrospective design, no information about the training sessions between the matches, and the number of players rotating. However, further investigations are needed to identify the specific injury circumstances and correlation to kick-off times and number of games. A thorough understanding of these factors may lead to the prevention of muscle and ligamentous injuries by modification of practice and kick-off times.

## Conclusion

This study exhibits a significant correlation between the three kick-off time groups and the lay-off times following muscle, ankle ligament, and knee ligament injury. The mean duration of lay-off after muscle injuries and ankle ligament was significantly longer when the injury was suffered in the night-group compared to the earlier kick-off group. Interestingly lay-off times for ligamentous injuries of the knee were shorter when the injury was suffered in the night-group. Reasons for these observations could be found in the circadian rhythms and muscle fatigue.

## Data Availability

The datasets generated and analyzed during this study are available from the corresponding author upon reasonable request.
